# Global improvement in right ventricular function after Stage II Norwood operation in children with hypoplastic left heart assessed by serial MRI

**DOI:** 10.1186/1532-429X-13-S1-P186

**Published:** 2011-02-02

**Authors:** Hannah R Bellsham-Revell, Reza Razavi, Philipp Beerbaum, Gerald Greil, Aaron Bell

**Affiliations:** 1 King's College London, London, UK; 2 Guy's and St Thomas' Foundation NHS Trust, London, UK

## Introduction

Hypoplastic left heart syndrome (HLHS) describes a spectrum of left heart hypoplasias, which leave the left ventricle unable to support the systemic circulation. Staged palliation consists of aortic reconstruction and systemic to pulmonary artery shunt in the neonatal period (stage I), followed at intervals by superior (Stage II) and then total cavopulmonary connection (Stage III). The initial operation results in a volume loaded systemic right ventricle (RV) which is reduced after Stage II. In our institution children are assessed prior to Stage II and Stage III with a cardiac magnetic resonance imaging (MRI) scan

## Methods

Ethical and institutional approval was obtained. MRI scans of children with HLHS assessed prior to Stage II and Stage III surgery were analysed. RV volumes and function were assessed and indexed to body surface are (BSA). Currently ‘normal’ function is taken as an ejection fraction (EF) of >50%. Children were divided into two groups: those with RV EF >50% on the pre-Stage II MRI and those with RV EF <50% on this scan.

## Results

**Figure 1:**
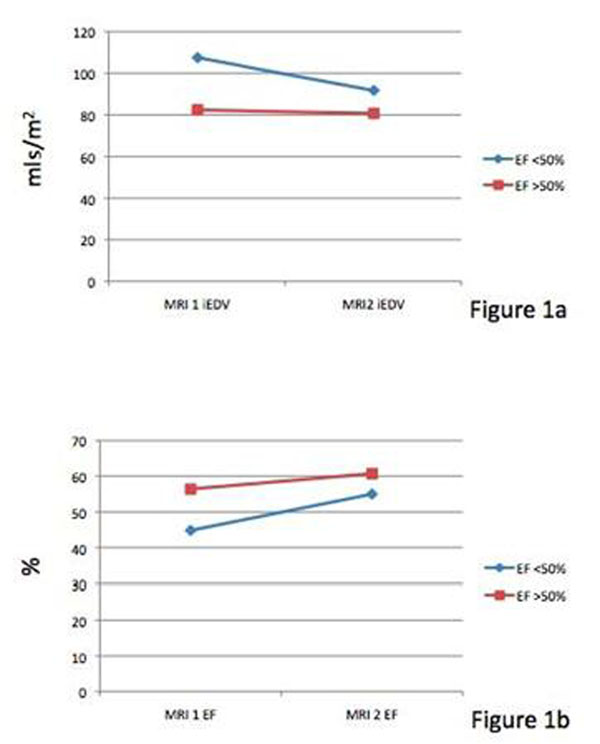


51 patients had an analysable MRI scan before Stage II and before Stage III. 21 patients had an RV EF 50%. Demographics are shown in Table 1. The RV indexed end diastolic volume (iEDV) fell in both groups from the first to the second MRI, but more in those with an RV EF <50% (figure 1a). RV indexed end systolic volume (iESV) remained similar in both groups. RV indexed stroke volume (iSV) increased in the RV EF 50% group. RV EF increased by 22.8% in the RV EF 50% group (figure 1b). The greatest increase in RV EF was seen in the group with an RV EF <50% who had Stage II at less than 6 months of age (figure 1b).

**Table 1:** 

	Median Age (years)	Median Weight (kg)	Median Height (cm)	Median BSA (m2)	Median Saturations (%)
MRI 1 <50%	0.343	5.5	61	0.308	74
MRI 1 >50%	0.465	6.34	64.5	0.341	76

## Conclusions

Median RV EF of the group with an RV EF <50% at the time of the pre-Stage II MRI scan increased to the normal range by the pre-Stage III MRI scan. An increase was also seen in the >50% group. This suggests that the normal ranges may need to be adapted for operative stage, due to the RV loading after the Stage I surgery.

